# Influence of Sex on Stroke Prognosis: A Demographic, Clinical, and Molecular Analysis

**DOI:** 10.3389/fneur.2019.00388

**Published:** 2019-04-17

**Authors:** Emilio Rodríguez-Castro, Manuel Rodríguez-Yáñez, Susana Arias, María Santamaría, Iria López-Dequidt, Ignacio López-Loureiro, Manuel Rodríguez-Pérez, Pablo Hervella, Tomás Sobrino, Francisco Campos, José Castillo, Ramón Iglesias-Rey

**Affiliations:** ^1^Clinical Neurosciences Research Laboratory, Department of Neurology, Clinical University Hospital, Health Research Institute of Santiago de Compostela (IDIS), Santiago de Compostela, Spain; ^2^Stroke Unit, Department of Neurology, Hospital Clínico Universitario, Santiago de Compostela, Spain

**Keywords:** blood-brain barrier, critical care, hemorrhage transformation, ischemic stroke, prognosis

## Abstract

Identifying the complexities of the effect of sex on stroke risk, etiology, and lesion progression may lead to advances in the treatment and care of ischemic stroke (IS) and non-traumatic intracerebral hemorrhage patients (ICH). We studied the sex-related discrepancies on the clinical course of patients with IS and ICH, and we also evaluated possible molecular mechanisms involved. The study's main variable was the patient's functional outcome at 3-months. Logistic regression models were used in order to study the influence of sex on different inflammatory, endothelial and atrial dysfunction markers. We recruited 5,021 patients; 4,060 IS (54.8% male, 45.2% female) and 961 ICH (57.1% male, 42.9% female). Women were on average 5.7 years older than men (6.4 years in IS, 5.1 years in ICH), and more likely to have previous poor functional status, to suffer atrial fibrillation and to be on anticoagulants. IS patients showed sex-related differences at 3-months regarding poorer outcome (55.6% women, 43.6% men, *p* < 0.0001), but this relationship was not found in ICH (56.8% vs. 61.9%, *p* = 0.127). In IS, women had higher levels of NT-proBNP and 3-months worse outcome in both cardioembolic and non-cardioembolic stroke patients. Stroke patients showed sex-related differences in pre-hospital data, clinical variables and molecular markers, but only IS patients presented independent sex-related differences in 3-months poor outcome and mortality. There was a relationship between the molecular marker of atrial dysfunction NT-proBNP and worse functional outcome in women, resulting in a possible indicator of increased dysfunction.

## Introduction

Stroke among adults is a common cause of death, disability and poor functional outcome in Europe and other developed countries. Due to the aging of the population, the incidence of cerebrovascular disease is increasing and it seems that it will continue to do so in the next decades (1, 2). In this situation, the demographic conditions (lifestyle habits and socio-economic status), therapeutics strategies, healthcare structures, as well as clinical and molecular variables are known to play an important role in stroke incidence, treatment, clinical course and patient mortality (3–5).

According to recent research, ischemic stroke (IS) and non-traumatic intracerebral hemorrhage (ICH) patients present sex-related differences in functional outcomes and mortality (6, 7). Biological factors, clinical variables, and little differences in stroke diagnosis and management could be the more important sex-related considerations (8–11). In particular, conventional stroke risk factors as hypertension, atrial fibrillation, diabetes mellitus and inflammation processes have recently received more sex differences attention (12, 13).

In this context, the study of sex-related differences and risks factors in the clinical results obtained should provide valuable data for a future personalized, safe and effective medical practice. To this end, we analyzed the data of the patients admitted to a specialized Stroke Unit of a tertiary hospital in Northwestern Spanish (Galicia) in the last 10 years. The main objective of this study was to evaluate the influence of sex in the functional outcomes in patients with IS and ICH. The secondary objective was to confirm whether sex differences were associated with an increase of inflammation, endothelial and atrial dysfunction markers during IS and ICH.

## Materials and Methods

### Study Design

From a prospective registry, we conducted a retrospective study enrolling patients with ischemic stroke and non-traumatic intracerebral hemorrhage admitted to the Stroke Unit (Neurology Department of the Hospital Clínico Universitario de Santiago de Compostela) and who were included in a prospective stroke registry (BICHUS). Recruitment period was from September 2007 to September 2017. This study was carried out in accordance with the Declaration of Helsinki of the World Medical Association (2008) and approved by the Ethics Committee of Clinical Research of Galicia (CEIC). Signed informed consent was obtained from every patient or their relatives after full explanation of the procedures. The inclusion criteria for the study was IS and ICH patients attended by a neurologist according to common protocol (14, 15) and admitted to the stroke unit, patients without subsequent diagnostic confirmation were excluded.

### Demographic, Clinical, and Molecular Variables

The pre-hospital variables analyzed were: age, history of arterial hypertension (at least 2 blood pressure measurements >140/85 mmHg or under antihypertensive treatment), diabetes (previous diagnosis or under anti-diabetic treatment), alcoholism (>300 g of alcohol per week), smoking (habitual smoker or up to the previous year), dyslipidemia (at least a previous determination of total cholesterol >230 mg/dL or anti-hyperlipidemic treatment), peripheral arterial disease, coronary disease, atrial fibrillation, known carotid disease, prior transient ischemic attack, history of IS or ICH, anticoagulants and antiplatelet agents.

The clinical variables collected were: National Institute of Health Stroke Scale (NIHSS) (16) on admission, at 48 h and at discharge, previous Modified Rankin Scale (mRS) (17), at discharge and at 3-months, latency time from the stroke onset, whether it was a wake-up stroke, axillary temperature at admission, admission systolic blood pressure (SBP), diastolic blood pressure (DBP), and reperfusion treatments (intravenous or intraarterial fibrinolysis, thrombectomy, or both procedures).

Blood sample measurements were: glucose, erythrocyte sedimentation rate (ESR), glycated hemoglobin, triglycerides, LDL and HDL cholesterol. The inflammation molecular markers included were leukocytes, fibrinogen and C-reactive protein. Microalbuminuria and N-terminal pro-B-type Natriuretic Peptide (NT-proBNP) were the endothelial and atrial dysfunction markers determined, respectively.

Neuroimaging variables in IS were: baseline infarct volume (DWI-lesion) and ischemic lesion volume in a second computed tomography (CT) between 4th and 7th day, existence and type of hemorrhagic transformation (according to ECASS II criteria) (18). In hemorrhagic stroke, we collected basal hematoma volume (CT) and hematoma and edema volume (second CT between 4th and 7th day). IS and ICH and perihematomal edema volumes were calculated by using the ABC/2 method (19). ICH topography was classified as lobar when it predominantly affected the cortical / subcortical white matter of the cerebral lobes, or as deep when it was limited to the internal capsule, the basal ganglia or the thalamus, brain stem and cerebellum. All neuroimaging evaluations were performed by senior neuroradiologists blinded to the clinical data.

The etiological diagnosis of IS was performed according to TOAST criteria (20). Non-traumatic ICH was classified into hypertensive, amyloid, related to anti-platelets/anticoagulants and undetermined (21, 22).

The mRS at 3 ± 1 months was assessed face-to-face or by telephone by accredited expert neurologists (ER-C, IL-D, MS-C, SA-R, MR-Y). The computed tomography study performed at the 4th−7th was performed in 3,878 of the total patients included (79.39% IS and 68.5 ICH). Demographic and clinical data were available of all patients, laboratory variables microalbuminuria in 2,760 patients and NT-proBNP levels in 3,372.

### Primary Endpoint

The study's primary endpoint was the functional outcome evaluated by mRS at 3-months after stroke. We considered good functional outcome a mRS score of ≤2 and poor functional outcome or morbidity a mRS>2. Early neurological deterioration was defined as an increase of ≥4 points in NIHSS within the first 48 h with respect to baseline NIHSS score. We also analyzed the association between different biomarkers (inflammation, endothelial and atrial dysfunction) and outcome variables in IS and ICH patients.

We defined hospital improvement as the percentage difference between the basal and discharge NIHSS through the formula ([NIHSS at admission—NIHSS at discharge]/NIHSS at admission × 100), and the outpatient improvement between the NIHSS at discharge and at 3 ± 1 months, using the formula ([NIHSS at discharge—NIHSS at 3-months]/NIHSS at discharge × 100).

Sex possible correlations between NT-proBNP serum levels and 3-months modified Rankin Scale (mRS) score after stroke were evaluated in all patients, cardioembolic and non-cardioembolic stroke patients, as well as in early neurological deterioration patients. A possible relationship between hematoma growth and endothelial dysfunction marker were studied in all patients, female, male and male with ICH of hypertensive etiological origin.

### Statistical Analyses

Results were expressed as percentages for categorical variables and as mean [standard deviation (SD)] or median and range (25th−75th percentiles) for continuous variables depending on whether their distribution was normal or not. The Kolmogorov-Smirnov test was used to assess normality. Proportions were compared using the chi-square test. The Student's *t*-test (normal data) and the Mann Whitney test (non-normal data) was used to compare continuous variables. Correlations were performed using Pearson's (normally distributed variables) or Spearman (variables without normal distribution) coefficients. Each logistic regression analysis model (Enter method) was adjusted for the main baseline variables with a proven relevance for outcome in the univariate analyses and taking into account the clinical relevance. Results were expressed as odds ratios (ORs) with 95% confidence intervals (CI 95%). *p* < 0.05 was considered to be statistically significant in all tests. Statistical analysis was conducted in SPSS 21.0 (IBM, USA).

## Results

We recruited 5,021 patients; 4,060 were IS and 961 were ICH. Of these IS, 2,226 (54.8%) were male and 45.2% (1,834) were female; the mean male and female ages were 68.7 ± 13.1 and 75.1 ± 14.4. Of ICH patients, 549 (57.1%) were male and 412 (42.9%) female; the mean male and female ages were 71.1 ± 12.8 and 76.2 ± 13.1.

### Sex-Related Analysis on IS Patients

Ischemic stroke differences between sexes are detailed in Table 1. Women were older, had a higher proportion of hypertension, ischemic heart disease, atrial fibrillation, and to be on anticoagulants. Men had greater smoking and alcohol use, had a higher proportion of previous ischemic heart and peripheral arterial disease.

**Table 1:** Univariate analysis according to sex in ischemic stroke patients (*n* = 4,060).

	**Male (*n* = 2,226)**	**Female (*n* = 1,834)**	***p-value***
**DEMOGRAPHIC VARIABLES**			
Age (years)	68.7 ± 13.1	75.1 ± 14.4	<0.0001
History of hypertension, %	59.1	67.7	<0.0001
History of diabetes mellitus, %	24.7	23.5	0.397
History of alcoholism, %	18.2	3.4	<0.0001
History of smoking, %	24.5	7.3	<0.0001
History of dyslipidemia, %	34.3	34.1	0.947
Peripheral arterial disease, %	7.9	2.9	<0.0001
Previous ischemic heart disease, %	13.7	8.6	<0.0001
Ischemic heart disease, %	3.5	5	0.023
Atrial fibrillation, %	14.2	25	<0.0001
Previous transient ischemic attack, %	5.6	4.8	0.258
History of Ischemic stroke, %	14.2	14	0.982
History of Intracerebral hemorrhage, %	1	1.1	0.982
Anticoagulants, %	7.1	9.3	0.011
Antiaggregants, %	23.9	24.9	0.509
**CLINICAL VARIABLES**			
Stroke on awakening, %	9.6	10.3	0.460
Previous mRS	0 [0, 0]	0 [0, 1]	<0.0001
Time from stroke onset (minutes)	222.5 ± 167.4	256.5 ± 169.1	<0.0001
NIHSS score at admission	13 [7, 18]	15 [9, 21]	<0.0001
Early neurological deterioration, %	9.0	9.7	0.408
TOAST			<0.0001
Atherothrombotic, %	27.1	21.2	
Cardioembolic, %	32.2	40.7	
Lacunar, %	8.3	6.7	
Undetermined, %	30.8	31.0	
Others, %	1.5	0.5	
Intravenous fibrinolysis, %	21.3	22.9	0.223
Thrombectomy, %	3.4	3.3	0.861
DWI at admission (mL)	53.4 ± 106.4(170)	51.8 ± 98.1(132)	0.893
CT volume 4th−7th day (mL)	47.6 ± 79.6	63.8 ± 94.8	<0.0001
Hemorrhagic transformation			0.026
IH1, %	5.3	7.0	
IH2, %	2.2	3.4	
PH1, %	1.1	1.5	
PH2, %	1.0	1.6	
Axillary temperature at admission (°C)	36.3 ± 0.6	36.3 ±0.6	0.584
Blood glucose at admission (mg/dL)	137 ± 56.3	134.7 ± 52.6	0.971
Sedimentation rate (mm)	22.1 ± 21.9	29.2 ± 23.3	<0.0001
Glycosylated hemoglobin, %	6.3 ± 3.7	6.1 ± 1.2	0.185
SBP at admission (mmHg)	161.1 ± 31.9	162.9 ± 32.1	0.593
DBP at admission (mmHg)	87.1 ± 18.5	88.2 ± 19.6	0.318
LDL cholesterol (mg/dL)	110.3 ± 39.1	108.1 ± 36.5	0.598
HDL cholesterol (mg/dL)	39.1 ± 17.2	45.2 ± 16.5	<0.0001
Triglycerides (mg/dL)	124.6 ± 70.4	112.3 ± 56.3	<0.0001
**MOLECULAR MARKERS**			
Leukocytes at admission (× 10^3^/mL)	8.9 ± 3.1	9.6 ± 3.3	<0.0001
Fibrinogen at admission, mg/dL	431.4 ± 99.4	453.7 ± 98.7	<0.0001
C-reactive protein at admission (mg/dL)	2.7 ± 3.8	3.7 ± 4.2	<0.0001
Microalbuminuria (mg/24 h)	7.9 ± 26.2	7.0 ± 25.9	0.633
NT-proBNP levels (pg/mL)	915.9 ± 1563.7	1544.1 ± 1978.7	<0.0001
**OUTCOME**			
mRS at 3-months	2 [0, 4]	3 [1, 4]	<0.0001
Poor outcome, %	43.6	55.6	<0.0001

According to clinical variables, women had a higher proportion of previous poor functional status [0 [0, 1] vs. 0 [0, 0], *p* < 0.0001] and higher NIHSS at admission [13 [7, 18] vs. 15 [9, 21], *p* < 0.0001]. As to early neurological deterioration and stroke on awakening, there were no significant differences associated to sex among patients.

There were sex-related differences in the latency time from the stroke onset and care in the Emergency Department (222.5 ± 167.4 min in men, 256.5 ± 169.1 min in women; *p* < 0.0001).

There were significant sex-related variations regarding the IS subtype (*p* < 0.0001), where the high percentage of cardioembolic strokes (40.7% vs. 32.2%) is worthy of attention.

No sex differences were found among patients who received r-tPA and thrombectomy. However, sex differences were found in hospital and outpatient improvement of IS patients with or without fibrinolytic treatment. Women with fibrinolytic therapy showed substantial hospital improvement (71.3 ± 34.1% of men, 81.4 ± 28.3% of women; *p* < 0.0001) and this tendency seemed to be reversed regarding outpatient improvement (44.6 ± 42.9% of men, 41.1 ± 50.1% of women; *p* = 0.271). While all groups of patients without fibrinolytic treatment did not seem to vary their hospital improvement (17.2 ± 34.1% of men, 15.9 ± 37.3% of women; *p* = 0.348), female got worse at discharge (19.1 ± 55.2% of men, 6.8 ± 50.4% of women; *p* < 0.0001).

There were no sex differences in DWI at admission. However, the computed tomography scan performed at 4th−7th day reflected sex differences in the lesion volume (47.6 ± 79.6 mL in men, 63.8 ± 94.8 mL in women; *p* < 0.0001) and hemorrhagic transformation (IH1 5.3% vs. 7.0%). Women had higher levels of sedimentation rate and HDL cholesterol.

Molecular markers showed that female patients had higher levels of inflammation markers (leukocytes, fibrinogen, C-reactive protein) and atrial dysfunction marker (NT-proBNP levels). Despite that, the inflammatory response was slightly higher in women; there were no sex differences in their 3-months mRS.

**Figure 1:**
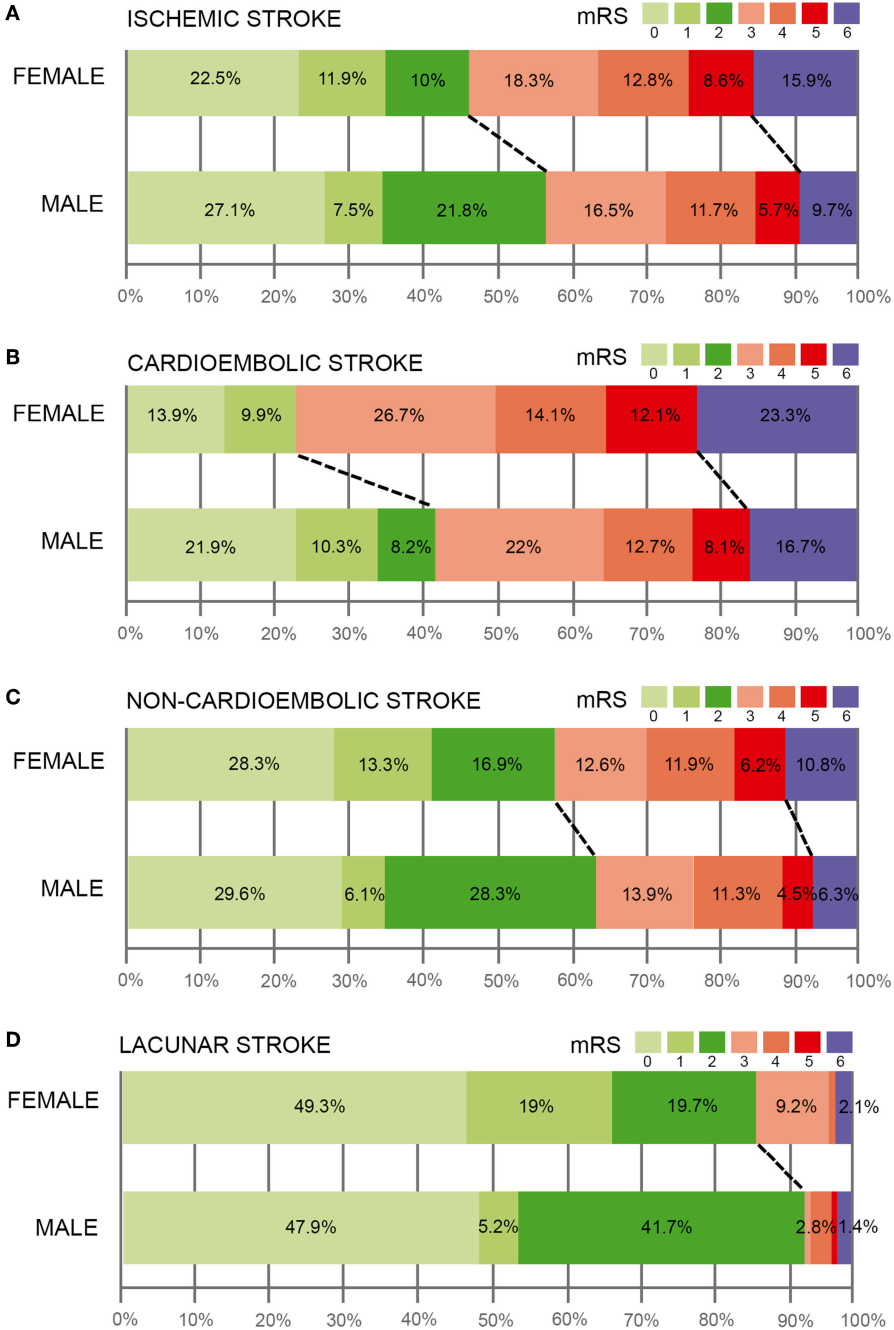
Modified Rankin Scale (mRS) scores in ischemic stroke patients **(A)**, cardioembolic **(B)**, and non-cardioembolic **(C)** stroke patients categorized by sex. **(D)** distribution of mRS scores in lacunar IS.

Figures 1A–C shows the distribution of mRS scores in all IS patients, cardioembolic stroke and non-cardioembolic stroke patients categorized by sex. In the three groups analyzed, a good functional outcome at 3-months was more frequent among males (IS: 56.4% of men vs. 44.4% of women; cardioembolic stroke: 40.4% of men vs. 23.8% of women; non-cardioembolic stroke: 64% of men vs. 58.5% of women). Interestingly, sex-related differences in 3-months functional outcome were observed, although women had a higher proportion of poor functional status in the case of cardioembolic strokes. Indeed, when we analyzed the relationship with morbidity and mortality in the three groups, cardioembolic strokes also presented positive sex-related variations in both variables. This highlights that non-cardioembolic stroke exposed differences only in mortality. Figure 1D shows the distribution of mRS scores in lacunar IS, where a good functional outcome at 3-months was more frequent among males (88% of men vs. 94% of women).

**Figure 2:**
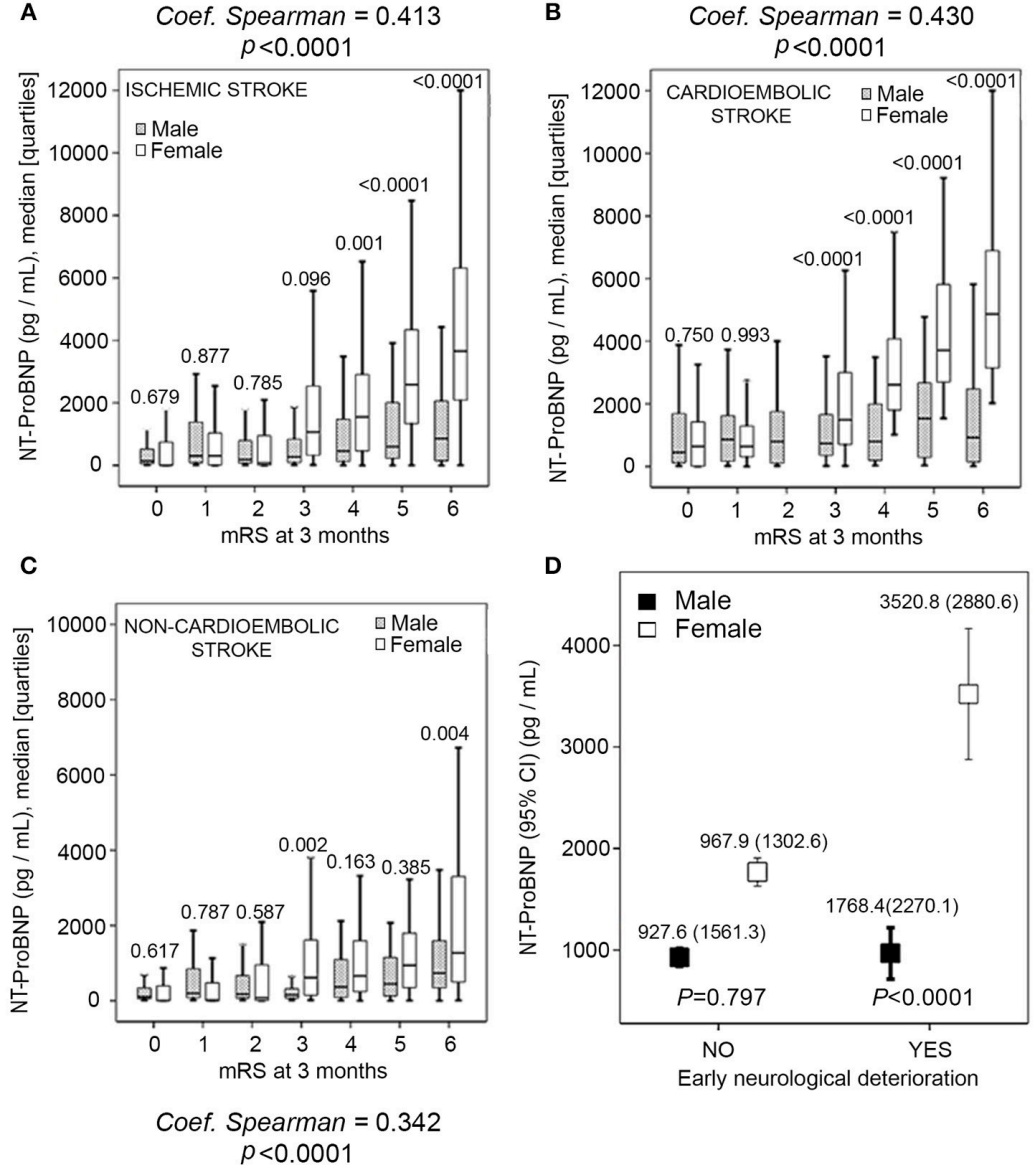
**(A–C)** Sex correlations between pro-brain natriuretic peptide (NT-proBNP) serum levels and 3-months modified Rankin Scale (mRS) score after stroke in all patients, cardioembolic and non-cardioembolic stroke patients. **(D)** Sex-related difference in NT-proBNP levels with and without early neurological deterioration patients.

The univariate analysis showed that women had higher levels of NT-proBNP. Female cardioembolic stroke with higher NT-proBNP concentration had worse, 3-months outcome, while in the non-cardioembolic stroke this tendency seemed to be less marked (Figures 2A–C). In keeping with these results, we also found a clear sex-related difference in NT-proBNP levels among patients with early neurological deterioration (Figure 2D).

Finally, women had a higher proportion of 3-months poor functional outcome (55.6% vs. 43.6%, *p* < 0.0001) with higher mRS [3 [1, 4] vs. 2 [0, 4], *p* < 0.0001].

### Sex-Related Analysis of ICH Patients

Non-traumatic intracerebral hemorrhage differences between sexes are detailed in Table 2. Women were older than men (average 76.2 ± 13.1 vs. 71.1 ± 12.8 years, *p* < 0.0001), had a higher proportion of previous transient ischemic attack, atrial fibrillation and to be on anticoagulants. Men had greater smoking and alcohol use, and had a higher proportion of previous ischemic heart and peripheral arterial disease.

**Table 2:** Univariate analysis according to sex in ICH patients (*n* = 961).

	**Male (*n* = 549)**	**Female (*n* = 412)**	***p-value***
**DEMOGRAPHIC VARIABLES**			
Age (years)	71.1 ± 12.8	76.2 ± 13.1	<0.0001
History of hypertension, %	59.0	61.9	0.387
History of diabetes mellitus, %	21.5	18.7	0.293
History of alcoholism, %	22.0	5.8	<0.0001
History of smoking, %	15.8	3.9	<0.0001
History of dyslipidemia, %	34.1	38.8	0.136
Peripheral arterial disease, %	6.4	1.5	<0.0001
Previous ischemic heart disease, %	11.1	4.4	<0.0001
Ischemic heart disease, %	2.7	4.1	0.233
Atrial fibrillation, %	15.1	20.4	0.033
Previous transient ischemic attack, %	1.5	3.2	0.075
History of Ischemic stroke, %	7.8	9.7	0.509
History of Intracerebral hemorrhage, %	9.1	10	0.509
Anticoagulants, %	11.5	16.5	0.025
Antiaggregants, %	16.6	14.8	0.457
Stroke on awakening, %	4.9	4.4	0.759
**CLINICAL VARIABLES**			
Previous mRS	0 [0, 1]	1 [0, 1]	<0.0001
Time from stroke onset (minutes)	212.7 ± 198.2	285.4 ± 256.2	<0.0001
NIHSS score at admission	12 [7, 16]	13 [8, 18]	0.003
Early neurological deterioration, %	27.3	26.9	0.942
Etiology			0.008
Hypertensive, %	50.6	46.6	
Amyloid, %	4.9	9.0	
Anticoagulants, %	11.7	16.3	
Others / Undetermined, %	32.8	28.2	
Hematoma volume at admission (mL)	42.1 ± 37.6	41.2 ± 32.8	0.934
Hematoma volume 4th−7th day (mL)	46.2 ± 43.5	45.2 ± 35.5	0.767
Total hematoma volume (mL)	61.5 ± 52.4	58.0 ± 41.4	0.418
Volume of hypodensity (mL)	15.2 ± 20.1	12.8 ± 15.4	0.129
Hematoma growth (mL)	14.1 ± 30.9	6.4 ± 26.1	0.001
Topography			0.389
Deep hemispheric, %	53.8	48.1	
Lobar, %	35.2	41.5	
Cerebellar, %	5.1	4.9	
Breinstem, %	3.8	3.9	
Primary intraventricular, %	2.0	1.7	
Axillary temperature at admission (°C)	36.5 ± 0.7	36.4 ± 0.7	0.276
Blood glucose at admission (mg/dL)	129.6 ± 38.1	137.7 ± 49.21	0.220
Sedimentation rate (mm)	22.5 ± 19.8	26.9 ± 23.4	0.904
Glycosylated hemoglobin, %	5.7 ± 0.8	5.8 ± 0.9	0.909
SBP at admission (mmHg)	167.4 ± 42.1	165.8 ± 39.6	0.612
DBP at admission (mmHg)	86.2 ± 20.5	89.3 ± 21.6	0.403
LDL cholesterol (mg/dL)	106.5 ± 35.3	115.9 ± 38.0	0.021
HDL cholesterol (mg/dL)	37.1 ± 19.7	40.8 ± 19.6	<0.0001
Triglycerides (mg/dL)	96.6 ± 46.8	105.2 ± 43.1	0.303
**MOLECULAR MARKERS**			
Leukocytes at admission (×10^3^/mL)	8.7 ± 3.4	9.0 ± 2.8	0.308
Fibrinogen at admission, mg/dL	422.9 ± 92.7	468.2 ± 99.8	<0.0001
C-reactive protein at admission (mg/dL)	5.0 ± 5.1	5.1 ± 4.5	0.300
Microalbuminuria (mg/24 h)	20.8 ± 38.3	16.1 ± 21.9	0.131
NT-proBNP levels (pg/mL)	850.6 ± 1235.5	934.8 ± 1508.08	0.229
**OUTCOME**			
mRS at 3-months	3 [1, 6]	3 [2, 6]	0.078
Poor outcome, %	56.8	61.9	0.127

As to clinical variables, women had a higher proportion of previous poor functional status [0 [0, 1] vs. 1 [0, 1], *p* < 0.0001] and higher NIHSS at admission [13 [8, 18] vs. 12 [7, 16], *p* = 0.003]. Like in the case of IS, there were no significant sex differences among ICH patients as regards early neurological deterioration and stroke on awakening.

We found sex-related variations in the latency time from the stroke onset and care in the Emergency Department (212.7 ± 198.2 min in men, 285.5 ± 156.2 min in women; *p* < 0.0001).

When analyzing patients according to the etiology of bleeding, there were significant sex differences (*p* = 0.008): hypertensive and undetermined hemorrhages were more frequent in males (50.6% vs. 46.6%; 32.8% vs. 28.2%, respectively), and cerebral amyloid angiopathy and hemorrhages by anticoagulants were more frequent in females (9.0% vs. 4.9%; 16.3% vs. 11.7%, respectively).

Although we found no sex differences in hematoma location (*p* = 0.389), there was a clear sex-related difference in lobar (35.2% of men, 41.5% of women) or deep ICH location (53.8% of men, 48.1% of women).

**Figure 3:**
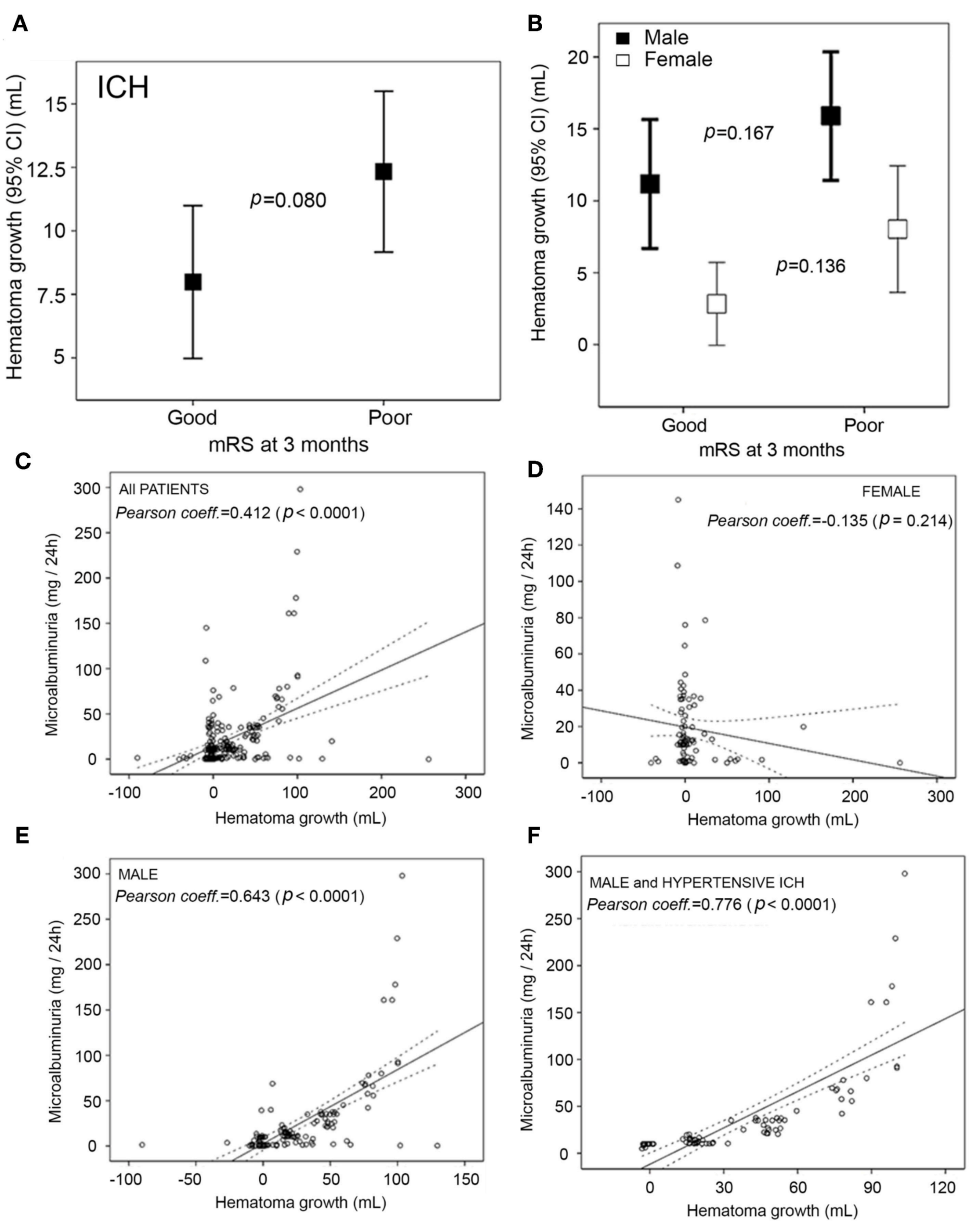
**(A)** Hematoma growth in good and poor outcome patients. **(B)** Sex-related difference in hematoma growth in 3-months good and poor outcome patients. **(C–F)** Correlation between hematoma growth and endothelial dysfunction marker in all patients, female, male and male with intracerebral hemorrhage of hypertensive etiological origin.

There were no differences between women and men in hematoma volume at admission and at 4th−7th day, although we found sex-related variations in the hematoma growth (14.1 ± 30.9 mL in men, 6.4 ± 26.1 mL in women; *p* = 0.001). Figures 3A,B details the hematoma growth by 3-months neurologic outcome. We found a statistically significant association when we analyzed the whole ICH sample, but this difference disappeared after adjusting for sex. In this line, we studied the association between hematoma growth and an endothelial dysfunction marker in all patients, female, male and male with ICH of hypertensive etiological origin. In Figures 3C–F a higher correlation can be seen in all groups except in females, i.e., all patients (*r* = 0.412, *p* < 0.0001), female (*r* = −0.135 *p* = 0.214).

Molecular markers indicated that women only presented higher levels of fibrinogen, while leukocytes, C-reactive protein and NT-proBNP levels were similar in both sexes.

**Figure 4:**
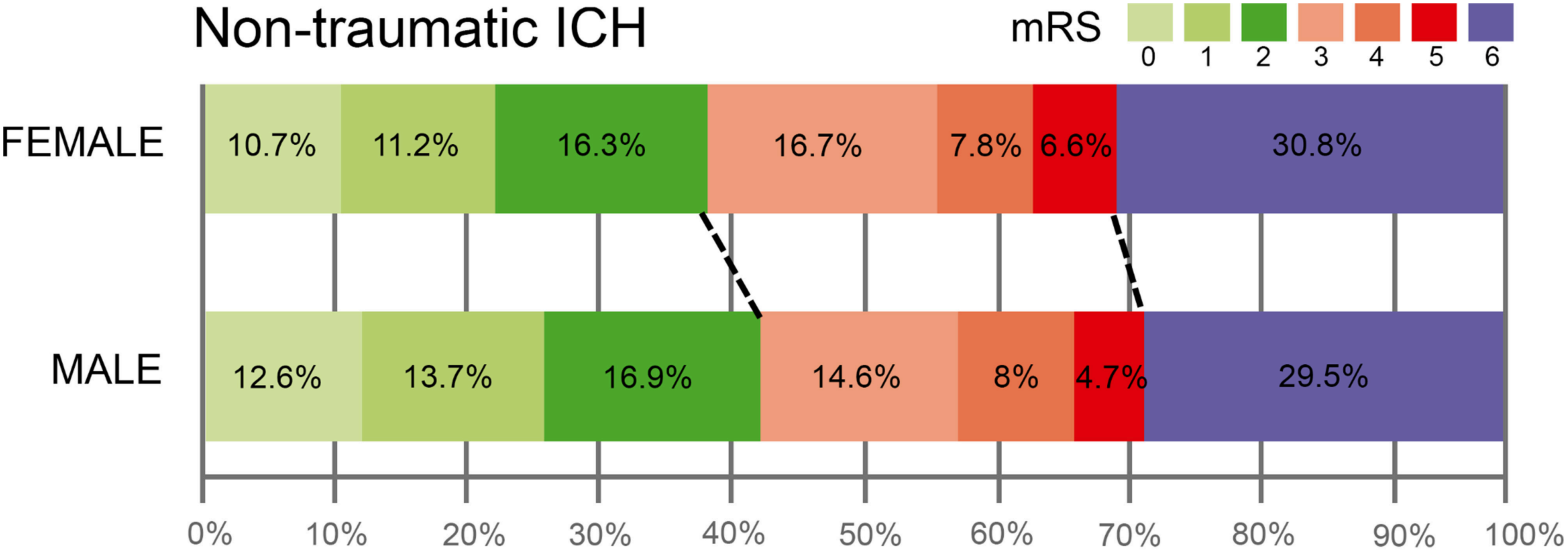
Distribution of the modified Rankin Scale (mRS) scores in ICH patients categorized by sex.

Finally, there were no sex differences in 3-months poor functional outcome (61.9% vs. 56.8%, *p* = 0.127). We saw, however, higher mRS [3 [2, 6] vs. [1, 6], *p* = 0.078]. Figure 4 shows the distribution of mRS scores in ICH patients, where 3-months good functional outcome was more frequent in males (43.2% of men vs. 38.2% of women). In keeping with these results, women were not independently associated with poor outcome in the logistic regression model (OR, 1.23; 95% CI, 0.95–1.60, *p* = 0.114).

### Baseline Characteristics of IS and ICH Patients by Neurologic 3-Months Outcome

Table 3 details the baseline demographic variables, clinical characteristics and molecular markers by 3-months neurologic outcome in IS. In summary, a good outcome was observed in 2,071 patients (51.0%) and a poor outcome was observed in 1,989 (49%). Patients with unfavorable outcome were older, smoked more, were more likely to be women, tended to present later to hospital after the onset of stroke, to suffer from hypertension, atrial fibrillation and ischemic heart disease. They had higher NIHSS scores, previous poor functional status, higher early neurological deterioration, higher axillary temperature at admission, higher blood glucose levels, hypercholesterolemia, higher sedimentation rate, higher frequency cardioembolic stroke, higher lesion volume (DWI at admission and CT 4th−7th day), to suffer hemorrhagic transformation and were under fibrinolytic treatment.

**Table 3:** Baseline demographic data, clinical characteristics, and molecular markers findings in IS patients with good or poor outcome at 3-months (*n* = 4,060).

	**Good outcome (*n* = 2,071)**	**Poor outcome (*n* = 1,989)**	***p-value***
**DEMOGRAPHIC VARIABLES**			
Age (years)	69.4 ± 12.3	76.8 ± 13.5	<0.0001
Female, %	36.7	55.3	<0.0001
Time from stroke onset (minutes)	227.7 ± 193.3	258.8 ± 154.3	0.001
History of hypertension, %	60.5	66.1	<0.0001
History of diabetes, %	23.4	25.1	0.210
History of smoking, %	21.1	11.3	<0.0001
History of alcoholism, %	11.2	11.9	0.459
History of hyperlipidemia, %	33.3	35.3	0.173
Atrial fibrillation, %	10.7	29.2	<0.0001
Previous ischemic heart disease, %	10.4	12.7	0.023
Ischemic heart disease, %	2.6	6.2	<0.0001
**CLINICAL VARIABLES**			
NIHSS at admission	16 [10, 20]	17 [11, 20]	<0.0001
Previous mRS	0 [0, 0]	0 [0, 1]	<0.0001
Axillary temperature at admission (°C)	36.3 ± 0.6	36.2 ± 0.5	<0.0001
Early neurological deterioration, %	2.3	17.0	<0.0001
Glucose levels (mg/dL)	158.3 ± 61.9	118.2 ± 34.6	<0.0001
LDL cholesterol (mg/dL)	106.6 ± 41.2	101.3 ± 31.3	0.002
HDL cholesterol (mg/dL)	44.5 ± 20.8	42.6 ± 15.7	0.273
Triglycerides (mg/dL)	113.5 ± 55.9	89.5 ± 36.6	<0.0001
Sedimentation rate (mm)	22.5 ± 22.0	28.6 ± 23.4	<0.0001
Glycosylated hemoglobin, %	6.6 ± 1.7	5.9 ± 1.3	0.978
TOAST- Cardioembolic stroke, %	18.4	57.2	<0.0001
DWI at admission (mL)	20.9 ± 26.4	43.6 ± 84	<0.0001
Fibrinolytic treatment, %	24.6	18.8	<0.0001
Thrombectomy, %	3.8	2.8	0.061
CT 4th−7th day (mL)	15.9 ± 20.8	34.5 ± 77.9	<0.0001
Hemorrhagic transformation, %	6.0	17.6	<0.0001
**MOLECULAR MARKERS**			
Leukocytes (×10^3^/mL)	8.7 ± 2.8	9.9 ± 3.5	<0.0001
Fibrinogen (mg/dL)	424.2 ± 93.6	462.9 ± 103.2	<0.0001
C-reactive protein (mg/L)	2.2 ± 3.4	4.4 ± 4.4	<0.0001
Microalbuminuria (mg/24 h)	7.6 ± 28.6	7.5 ± 22.4	0.964
NT-proBNP levels (pg/mL)	792.6 ± 1459.4	1702 ± 2018.3	<0.0001

In the multivariate model analysis, females were associated independently with the poor prognosis in all the models in which the demographic, clinical and molecular variables were introduced (Table 4). In Table 5 we detailed the ICH patient's characteristics by 3-months neurologic outcome. A good outcome was observed in 398 patients (41.4%) and a poor outcome was observed in 563 (58.6%). Patients with unfavorable outcome were older, had a higher proportion of atrial fibrillation and ischemic heart disease, but there were no sex differences. It should be highlighted that the molecular markers associated with a poor outcome were the ones corresponding with inflammation and endothelial dysfunction.

**Table 4:** Multivariate analysis: Adjusted OR of poor outcome at 3-months for baseline associated variables in the univariate analysis in IS patients.

**Independent variables**	**OR**	**95% CI**	***p*-value**
Female not adjusted	2.13	1.88–2.42	<0.0001
**DEMOGRAPHIC VARIABLES**			
Female	1.54	1.32–1.80	<0.0001
Age	1.03	1.02–1.04	<0.0001
Previous mRS	1.39	1.19–1.61	<0.0001
History of atrial fibrillation	2.18	1.79–2.67	<0.0001
Time from stroke onset	1.00	1.00–1.00	0.025
**CLINICAL VARIABLES**			
Female	2.17	1.64–2.87	<0.0001
NIHSS at admission	1.06	1.03–1.08	<0.0001
Axillary temperature at admission	1.38	1.09–1.75	0.007
Glucose levels at admission	1.00	1.00–1.01	0.012
Sedimentation rate	1.00	1.00–1.01	0.004
Cardioembolic stroke	4.62	3.44–6.21	<0.0001
TC volume 4th−7th day	1.02	1.02–1.03	<0.0001
Fibrinolytic treatment	0.21	0.15–0.3	<0.0001
Early neurological deterioration	4.56	2.58–8.06	<0.0001
**MOLECULAR MARKERS**			
Female	1.91	1.57–2.31	<0.0001
Leukocytes	1.06	1.02–1.09	0.001
Fibrinogen	1.00	1.00–1.01	0.006
C-reactive protein	1.09	1.06–1.12	<0.0001
NT-proBNP levels (pg/mL)	1.00	1.00–1.00	<0.0001

**Table 5:** Baseline pre-hospital data, clinical characteristics and molecular markers findings in ICH patients with good or poor outcome at 3-months (*n* = 961).

	**Good outcome (*n* = 398)**	**Poor outcome (*n* = 563)**	***p-value***
**DEMOGRAPHIC VARIABLES**			
Age (years)	69.9 ± 13.5	76.0 ± 12.1	<0.0001
Female, %	40.3	45.6	0.054
Time from stroke onset (minutes)	208.3 ± 227.8	220.3 ± 209.2	0.669
History of hypertension, %	59.2	61.8	0.218
History of diabetes, %	17.9	21.6	0.084
History of smoking, %	12.6	8.8	0.059
History of alcoholism, %	15.8	15.4	0.468
History of hyperlipidemia, %	38.2	35.6	0.223
Atrial fibrillation, %	13.1	21.1	0.001
Previous ischemic heart disease, %	7.4	9.0	0.233
Ischemic heart disease, %	2.9	3.9	0.244
**CLINICAL VARIABLES**			
NIHSS at admission	11 [8, 14]	15 [10, 18]	<0.0001
Previous mRS	0 [0, 1]	1 [0, 1]	<0.0001
Axillary temperature at admission (°C)	36.3 ± 0.6	36.6 ± 0.9	<0.0001
Early neurological deterioration, %	6.9	25.7	<0.0001
Glucose levels (mg/dL)	126.3 ± 36.6	140.9 ± 55.7	<0.0001
LDL cholesterol (mg/dL)	115.1 ± 32.5	114.5 ± 38.1	0.376
HDL cholesterol (mg/dL)	38.3 ± 15.8	38.6 ± 26.5	0.967
Triglycerides (mg/dL)	112.9 ± 57.7	107.2 ± 45.7	0.193
Sedimentation rate (mm)	22.5 ± 22.0	28.6 ± 23.4	<0.0001
Glycosylated hemoglobin, %	5.7 ± 0.8	5.8 ± 0.9	0.092
Etiology			< 0.0000
Hypertensive, %	44.1	55.9	
Amyloid, %	19.4	80.6	
Anticoagulants, %	25.7	74.3	
Others/Undetermined, %	49.4	50.6	
Edema volume at admission (mL)	0.6 ± 24.6	2.6 ± 25.4	<0.0001
Hematoma growth (mL)	49.8 ± 28.9	53.6 ± 16.8	0.199
**MOLECULAR MARKERS**			
Leukocytes (×10^3^/mL)	7.5 ± 2.2	9.1 ± 3.5	0.002
Fibrinogen (mg/dL)	416.2 ± 64.0	471.7 ± 90.5	<0.0001
C-reactive protein (mg/L)	4.1 ± 3.1	6.3 ± 5.5	<0.0001
Microalbuminuria (mg/24 h)	25.1 ± 51.8	36.3 ± 34.2	0.018
NT-proBNP levels (pg/mL)	539.8 ± 890.7	673.2 ± 650.1	0.174

## Discussion

Of the whole sample analyzed in our study, women were on average 5.7 years older than men (6.4 years in IS, 5.1 years in ICH), more likely to have previous poor functional status, to suffer atrial fibrillation and to be on anticoagulants. These results may directly influence comorbidity, the intensity of neurological involvement, access to certain treatments or management protocols, and patient clinical course. In addition, as the aging of population is expected to continue, the patients with IS who will not be able to benefit from reperfusion treatment will increase, and the absence of an effective ICH treatment will aggravate this perspective. As to IS and ICH patients, our data suggest that only IS women had a higher proportion of 3-months poor functional outcome. Early neurological deterioration, morbidity and mortality were similar for the two sexes. Results are in agreement with recent findings related to the sex of the patient and post-stroke health outcome (7, 23).

Our study showed that women had later hospital arrival time compared with men from stroke onset (34 min in IS, 72.7 min in ICH). However, as it can be seen in the global results presented, arrival time is not likely to be a major contributor to differences in outcome and mortality between sexes, in keeping with a recent study (24).

Cardioembolic and non-cardioembolic IS patients categorized by sex determined that 3-months good functional outcome results, morbidity and mortality were more frequent in males. Several molecular markers were evaluated to identify representative prognostic factors in acute IS. We selected molecular markers that are associated with inflammation (including leukocytes, fibrinogen and C-reactive protein), associated with endothelial dysfunction (microalbuminuria), as well as associated with atrial dysfunction (NT-proBNP levels). We found that the inflammatory response is slightly higher in women, but an association with functional poor outcome was not statistically significant. However, it is thus interesting to note that in a recent clinical study (25), it has been determined that there are age and sex-related differences in lipid profiles among IS patients. In this line, a clinical research work have shown that middle-aged females have a detrimental combination of elevated pro-inflammatory T-cells and decreased anti-inflammatory regulatory T-cells in adipose tissue, which may promote a pro-inflammatory milieu and contribute to increased cardiovascular disease (26). Finally, these studies underline microglia as an important determinant of sex differences under physiological conditions and in the injured brain (13, 27).

On the other hand, there were no sex differences in microalbuminuria levels in our study. In contrast, the analysis showed that women had higher levels of NT-proBNP, and in cardioembolic stroke higher NT-proBNP concentration had 3-months worse outcome. This correlation is less marked in others subtype of IS. These findings support previous data about sex risk factors (28–30), and with a recent hospital based study; in which women-specific trends over a 24-year period (1986–2009) were an increase in the patient's age, hypertension, atrial fibrillation and cardioembolic infarction, as well as a decrease mortality and length of hospitalization (31). However, to our knowledge, independent association of women 3-months poor functional outcome with NT-proBNP levels in IS patients has not been previously accurately evaluated.

There were no sex differences among patients who received r-tPA and thrombectomy. However, we found sex differences in treatment between patients who received fibrinolytic treatment and those who did not; women showed substantial hospital improvement when they received r-tPA and this tendency seemed to be reversed regarding outpatient improvement. All patients without fibrinolytic treatment did not seem to vary their hospital improvement, while female seemed to get worse at discharge. Results are in line with a recent study on the influence of sex in stroke thrombolysis, where no relevant sex discrepancies were determined in outcome after intra-arterial thrombectomy (32).

We determined that there were significant sex differences in ICH patients on the basis of the etiology of bleeding: hypertensive and undetermined hemorrhages were more frequent in males; and cerebral amyloid angiopathy and hemorrhages by anticoagulants were more frequent in females. We did not find the same relationship with the hematoma location. A relationship between hematoma growth and men was obtained in ICH. However, its association with the functional poor outcome was not statistically significant. The endothelial dysfunction marker evaluated correlated significantly with the hematoma growth (except in female group), particularly in male patients with hypertensive ICH. Controversies exist regarding sex differences in ICH variables, hematoma expansion and mortality (33–35). A recent study concluded that patients with ICH showed sex-related differences in clinical variables, hematoma location, but not in stroke care, 3-months mortality, or adjusted poor outcome (7). Another similar clinical research determined that men with ICH experience a higher risk of both expansion and early and late mortality (35). Both studies highlight the importance of providing new data and analysis, such as those in our study, to explore the discrepancies observed.

Our study has some weaknesses: First, we conducted a retrospective single-center study. Second, the health area studied belongs mainly to urban and coastal population, with little rural population. Three, a relatively short follow-up time of 3-months as a consequence of clinical guidelines. On the other hand, our study also shows some strengths; we focused the analysis on a large homogeneous population of IS and ICH patients, with a strong demographic, clinical and molecular characterization. The data were obtained and included by the same neurologists trained in cerebrovascular diseases and all patients were managed under the same protocol.

## Conclusion

Our data suggest that women who suffer from IS present with a poorer functional outcome than men at 3-months, regardless of other preclinical and clinical factors during the acute phase. These relationships seem to be mediated by atrial dysfunction and inflammation. The inflammatory response is slightly higher in women; however, there are no sex differences in their functional behavior. There is a probable relationship between the molecular marker of atrial dysfunction NT-proBNP and worse functional outcome in women, and the connection seems to be more important in cardioembolic stroke patients.

In patients with ICH, the 3-months poor functional outcome, mortality and morbidity are similar for the two sexes. The hematoma growth is higher in men, although their association with the functional poor outcome is not statistically significant. Microalbuminuria could be an early marker of hematoma growth, particularly in male patients with hypertensive ICH.

## Ethics Statement

This research was carried out in accordance with the Declaration of Helsinki of the World Medical Association (2008) and approved by the Ethics Committee of the Servizo Galego de Saúde. Informed consent was obtained from each patient or their relatives after full explanation of the procedures.

## Author Contributions

JC and RI-R participated in the design and coordination of the study, wrote the main manuscript text and analyzed the data. MR-Y, SA, MS, ER-C, and IL-D collected the data, have participated in the clinical assessment and manuscript preparation. IL-L and MR-P have been involved in the statistical analysis and manuscript preparation. PH, TS, and FC have been involved in revising the manuscript for important intellectual content and analysis. All authors have read the manuscript, agree that the work is ready for submission to a journal, and accept responsibility for the manuscript's content.

### Conflict of Interest Statement

The authors declare that the research was conducted in the absence of any commercial or financial relationships that could be construed as a potential conflict of interest. Hannah Roeder, Columbia University Irving Medical Center, New York, in collaboration with reviewer Eliza Miller
